# Infectious Bronchitis Virus Regulates Cellular Stress Granule Signaling

**DOI:** 10.3390/v12050536

**Published:** 2020-05-14

**Authors:** Matthew J. Brownsword, Nicole Doyle, Michèle Brocard, Nicolas Locker, Helena J. Maier

**Affiliations:** 1 The Pirbright Institute, Pirbright, Surrey GU24 0NF, UK; matthew.brownsword@pirbright.ac.uk (M.J.B.); nicole.doyle@pirbright.ac.uk (N.D.); 2 Faculty of Health and Medical Sciences, School of Biosciences and Medicine, University of Surrey, Guildford, Surrey GU2 7XH, UK; m.brocard@surrey.ac.uk (M.B.); n.locker@surrey.ac.uk (N.L.)

**Keywords:** infectious bronchitis virus, IBV, stress granule, SG, eIF2α, host shut-off, translation inhibition

## Abstract

Viruses must hijack cellular translation machinery to express viral genes. In many cases, this is impeded by cellular stress responses. These stress responses result in the global inhibition of translation and the storage of stalled mRNAs, into RNA-protein aggregates called stress granules. This results in the translational silencing of the majority of mRNAs excluding those beneficial for the cell to resolve the specific stress. For example, the expression of antiviral factors is maintained during viral infection. Here we investigated stress granule regulation by *Gammacoronavirus* infectious bronchitis virus (IBV), which causes the economically important poultry disease, infectious bronchitis. Interestingly, we found that IBV is able to inhibit multiple cellular stress granule signaling pathways, whilst at the same time, IBV replication also results in the induction of seemingly canonical stress granules in a proportion of infected cells. Moreover, IBV infection uncouples translational repression and stress granule formation and both processes are independent of eIF2α phosphorylation. These results provide novel insights into how IBV modulates cellular translation and antiviral stress signaling.

## 1. Introduction

During replication within a host cell, all viruses must regulate a variety of cellular processes to generate an environment that allows progeny virus to be produced to continue the infection cycle. This includes promoting pathways that are favorable to replication and overcoming intrinsic immune pathways. Cellular stress granules (SG) play an important role in the regulation of gene expression by regulating mRNA translation and location, as well as integrating intracellular signaling and antiviral responses, and are therefore often targeted by viruses [1,2]. SG are cytoplasmic, non-membrane bound aggregations of mRNA associated with translation initiation factors, the 40S ribosome and RNA binding proteins. They primarily form under stress conditions that trigger the phosphorylation of translation initiation factor eIF2α [3]. There are four eIF2α kinases; protein kinase R (PKR), recognizing dsRNA, PKR-like endoplasmic reticulum kinase (PERK), sensing ER stress, heme regulated eIF2α kinase (HRI) and general control nonderepressible 2 (GCN2), activated by oxidative stress and amino acid deprivation [4,5,6,7]. Despite PKR being the assumed major kinase to activate the integrated stress response (ISR) during viral infection, PERK [8] and GCN2 [9] have also been found to play an important role. Phosphorylation of eIF2α prevents delivery of the initiator tRNA to initiating ribosomes, therefore inhibiting translation initiation and leading to the accumulation of stalled 48S mRNPs. SG can also be formed independently of eIF2α by interference with the RNA helicase eIF4A, which is required to unwind the mRNA untranslated region during ribosome recruitment [10]. This can be achieved by use of the chemicals pateamine A [11], hippuristanol and hydrogen peroxide [12,13,14]. SG formation occurs in a multi-step process culminating in large compartments, with dense cores held together by weak RNA-protein interactions that can merge to form SG with multiple cores. This process is driven by interactions between aggregation prone RNA binding proteins, including Ras GAP SH3-domain binding protein 1 (G3BP1), T-cell restricted intracellular antigen 1 (TIA-1) and TIA-1 related protein (TIAR) [1,15,16]. Next, a liquid-like layer is formed around the core by liquid-liquid phase separation. This is achieved by interactions between RNA binding proteins containing intrinsically disordered regions (IDR) and by RNA-RNA interactions [16,17,18,19,20]. SG are highly dynamic, able to rapidly assemble, fuse and dissolve. They can act as storage sites for mRNAs, allowing either rapid translation reactivation upon stress resolution or the shuttling of mRNAs to sites of decay. SG are also proposed to play a role in antiviral signaling as key signaling proteins, including MDA5 and PKR, are known to localize to SG, and SG formation is involved in PKR activation [1,21].

Viruses rely on cellular translation machinery for the synthesis of viral proteins. Therefore, the role of SG in the inhibition of translation means they are often targeted by viruses to disrupt their function. Some viruses induce SG at early time points post infection but then inhibit their formation at later stages, either by inhibiting phosphorylation of eIF2α [22], or by cleaving SG scaffold proteins like G3BP1 [23]. Other viruses prevent formation of canonical SG by redirecting SG proteins to virus driven atypical granules that co-localize with sites of viral RNA synthesis or particle assembly, benefiting virus replication [24,25].

Coronaviruses are positive strand RNA viruses that cause economically important diseases in humans and other species, including porcine epidemic diarrhea virus, severe acute respiratory syndrome coronavirus (SARS-CoV), Middle East Respiratory Syndrome (MERS)-CoV and the recently emerged SARS-CoV-2. SARS-CoV, MERS-CoV and SARS-CoV-2 all emerged following cross species transmission events. It is important to understand how this family of viruses interact with the innate immune and stress responses of their hosts, which may inform the understanding of barriers to zoonotic transmission. Only a few studies have been performed on the role of SG during the replication of coronaviruses. SG were found in cells infected with *Alphacoronavirus* transmissible gastroenteritis virus (TGEV). Here, viral RNA was found to be targeted to SG, via an interaction with polypyrimidine tract binding protein (PTB) [26]. SG were also found in cells infected with *Betacoronavirus* mouse hepatitis virus (MHV). Knock down of SG components, such as G3BP1 or the prevention of eIF2α-phosphorylation, resulted in increased viral replication, suggesting SG or G3BP1 itself, perform an antiviral role [27]. In agreement with the latter, G3BP1 has been shown to attenuate viral replication independently of SGs [28,29]. Recently, *Betacoronavirus* MERS-CoV was found to inhibit SG formation via a process involving accessory protein 4a interaction with dsRNA and antagonism of PKR [30,31].

Infectious bronchitis virus (IBV) is a *Gammacoronavirus* causing infectious bronchitis, a respiratory disease in poultry responsible for reduced egg production and reduced meat quality. It has been shown by others that early during IBV infection, eIF2α is phosphorylated via both PKR and PERK activation. However, at later stages, eIF2α is dephosphorylated via the upregulation of GADD153 and GADD34, promoting activity of the phosphatase PP1 [32,33]. In addition, IBV has been shown to shut-off host translation in a process involving viral accessory protein 5b [34]. Despite this knowledge, the formation of SG or regulation of SG signaling during IBV replication and how this relates to regulation of translation has not been studied. Here, we present a detailed analysis of IBV regulation of cellular SG signaling and how this integrates with the shut-off of translation.

## 2. Materials and Methods

### 2.1. Cells, Viruses and Reagents

Vero cells were maintained in 1× Eagle’s modified essential medium (Sigma) supplemented with 1× l-glutamine (Gibco) and 10% fetal bovine serum (Sigma). Recombinant IBV strain Beau-R has been described previously [35]. Inactivated IBV was generated by treatment with binary ethylenimine (BEI). Briefly, the virus was incubated in 0.1 M BEI for 48 h at 37 °C, followed by inactivation of BEI by addition of 1 M sodium thiosulfate. Inactivation of virus was confirmed by RT-qPCR following the infection of cells. Sodium arsenite, cycloheximide, puromycin and emetine were purchased from Sigma.

### 2.2. Immunofluorescence

Vero cells seeded onto glass coverslips were mock infected or infected with undiluted IBV (MOI 0.02) and incubated at 37 °C. After 1 h, 1× BES (MEM, 0.3% tryptose phosphate broth, 0.2% bovine serum albumin, 20 mM *N*,*N*-Bis(2-hydroxyethyl)-2-aminoethanesulfonic acid (BES), 0.21% sodium bicarbonate, 2 mM l-glutamine, 250 U/mL nystatin, 100 U/mL penicillin, and 100 U/mL streptomycin) were added and cells were incubated for the indicated time. Where indicated, cells were treated for 1 h prior to fixation with 500 μM sodium arsenite or 35 µM cycloheximide, or for 2 h prior to fixation with 2 µM hydrogen peroxide. Cells were fixed in 4% paraformaldehyde in PBS, permeabilized in 0.1% triton X-100 in PBS and blocked in 0.5% bovine serum albumin (BSA) in PBS. Primary and secondary antibodies were diluted in blocking buffer. Nuclei were stained with 4′,6-diamidino-2-phenylindole (DAPI). Anti-dsRNA J2 (English and Scientific Consulting) was diluted 1:1000, anti-nsp12 [36] was diluted 1:1000, anti-S2 (26.1, Wageningen University and Research) was diluted 1:500, anti-IBV (Abcam) was diluted 1:1000, anti-G3BP1 (BD biosciences) was diluted at 1:500, anti-G3BP1 (Sigma) was diluted 1:500, anti-eIF3η (Santa-Cruz) was diluted 1:500 and anti-eIF4G (Santa-Cruz) was diluted 1:500. Alexa Fluor-conjugated secondary antibodies (Invitrogen) were diluted 1:500. Cells were visualized using a Leica SP5 or Nikon Ti Eclipse confocal microscope. To determine the percentage of the cells which were positive for SG, cells were counted manually, with at least 50 cells counted over three independent biological replicates.

### 2.3. Fluorescent in situ Hybridization (FISH)

Vero cells seeded onto glass coverslips were mock or IBV infected (MOI 0.02). After 24 h, cells were fixed and labelled using the Stellaris RNA FISH simultaneous labelling protocol (Biosearch technologies). Briefly, cells were fixed in 10% formaldehyde in PBS and permeabilized in 70% ethanol at 4 °C. Cells were incubated overnight at 37 °C in a humidified chamber, with hybridization buffer containing 125 nM probe and primary antibody. Cells were then washed and labelled with Alexa Fluor-conjugated secondary antibody and DAPI. Finally, cells were mounted onto glass coverslips using Vectashield and sealed with nail varnish. Cells were visualized using a Leica SP5 confocal microscope. Stellaris FISH probes with a Quasar 570 label were designed specifically for the nsp15 and nsp16 region of the IBV Beau-R genome.

### 2.4. Cell Lysis and Western Blot

Vero cells seeded in 6-well plates were mock infected or infected with undiluted IBV (MOI 0.005). At the indicated time points, cells were washed once with cold PBS and lysed in 1× sample buffer (Biorad) containing DTT. Cell lysates were heated to 95 °C for 3 min and briefly sonicated. Proteins were separated on a 4%–20% Bis-Tris gel (Biorad) and transferred onto nitrocellulose membranes. These were blocked in 0.5% BSA or 5% milk in TBS-Tween (TBS-T), then incubated with primary antibody diluted in blocking buffer. Following three washes in TBS-T, membranes were incubated with HRP labelled secondary antibodies (Dako), diluted in blocking buffer. After three further washes in TBS-T, blots are incubated chemiluminescence substrate using the Clarity Western ECL Substrate (Bio-Rad). Labelled protein bands were visualized, using a Vilber imaging system. Anti-IBV was diluted 1:1000, anti-eIF2α (Cell Signaling Technologies) was diluted 1:1000, anti-eIF2α-p (Cell Signaling Technologies) was diluted 1:2000 and anti-GAPDH (Invitrogen) was diluted 1:10000.

### 2.5. Ribopuromycylation (RPM)

Vero cells seeded onto glass coverslips were mock or IBV infected (MOI 0.02), as before. RPM was performed as described by David et al. [37] Briefly, one hour prior to processing, control wells were treated with 500 µM sodium arsenite. At the indicated times post infection, cells were incubated with 18.4 μM puromycin for 30 s at room temperature and then incubated with 18.4 μM puromycin and 208 μM emetine at room temperature for 1 min. Cells were washed three times with room temperature 1× BES media, fixed and processed for immunofluorescence, as described above. Anti-puromycin (Sigma) was diluted 1:10000. To quantify immunofluorescence images, the puromycin signal in 100 cells was determined using ImageJ [38].

## 3. Results

### 3.1. IBV Replication Induces Stress Granules in a Proportion of Infected Cells

**Figure 1:**
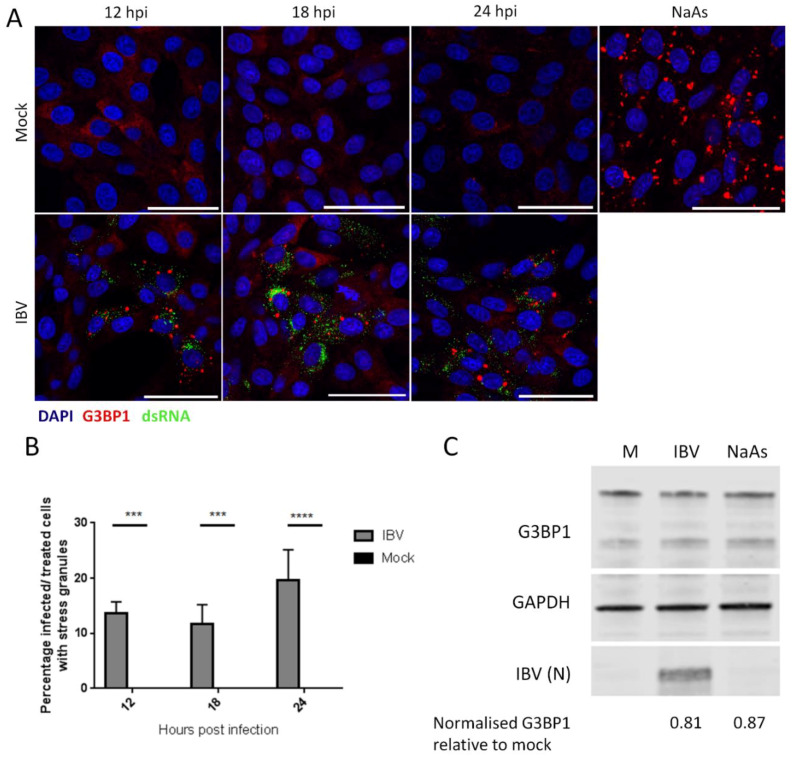
IBV (infectious bronchitis virus) infection induces stress granules in a proportion of infected cells. (**A**) Vero cells were mock infected or infected with IBV. At 12, 18 and 24 hpi, stress granules (SG) were detected using an anti-G3BP1 antibody (red) and IBV infection was detected with an anti-dsRNA antibody (green). Nuclei were stained with DAPI (blue). Positive control cells were treated with sodium arsenite (NaAs) to induce eIF2α-dependent SG. Scale bar indicates 50 μm. (**B**) Images in (**A**) were quantified by manual counting of SG positive cells, identified by counting infected or treated cells with G3BP1 foci. A minimum of 100 cells were counted from three independent replicates. The mean and standard deviation is shown. Asterisks indicate statistical significance, as measured by one-way ANOVA, *** represents *p* < 0.005 and **** represents *p* < 0.0005, respectively. (**C**) Vero cells were mock infected or infected with IBV. At 23 hpi, where indicated, cells were treated with NaAs. Cells were lysed at 24 hpi and processed and labelled using anti-G3BP1, anti-GAPDH or anti-IBV antibodies. Mean G3BP1 band intensities for IBV and NaAs treatments were normalized relative to mock band. Blot representative of 3 independent replicates.

Initially, the ability of IBV to induce SG during replication was assessed. Vero cells were infected with IBV and at the indicated time points, cells were fixed and labelled with anti-dsRNA to detect virus infection and with anti-G3BP1 to detect SG. At each time point, infected cells were present, with the number of infected cells increasing over time, as expected (Figure 1A). In addition, at each of the time points tested, G3BP1 puncta were detected in a proportion of, but not all, infected cells, with diffuse G3BP1 found in the remaining infected and uninfected cells. Subsequently, the number of infected cells with and without G3BP1 puncta was determined. The percentage of infected cells containing G3BP1 puncta was found to be between 10 and 25% (Figure 1B) and this percentage remained unchanged over the course of infection, with no statistical difference between the percentages of cells containing puncta at any time point. Therefore, IBV replication triggers the formation of G3BP1 puncta, but interestingly, only in 10%–25% of infected cells. Following identification of SG in IBV infected cells, the requirement for active virus replication in induction of granules was assessed. Cells were infected with wild type IBV or a BEI-inactivated virus. After 24 hours, cells were fixed and labelled with anti-S2 and anti-G3BP1. While cells infected with wild type IBV contained SG as observed before, cells infected with the inactivated virus did not (Figure S1). In addition, none of the compounds used for virus inactivation had any effect on SG formation or inhibition. Therefore, the induction of SG requires actively replicating virus and is not a response by the cell to the presence of the virus particle. Some viruses inhibit the formation of SG by degradation of SG nucleating proteins, such as G3BP1. Therefore, it was investigated whether G3BP1 is degraded during IBV infection. Cells were infected with IBV and after 24 h, cells were lysed, proteins separated by SDS-PAGE and levels of G3BP1, IBV N and GAPDH were measured by western blot (Figure 1C). G3BP1 levels were unaltered compared to mock treated cells upon either IBV infection or sodium arsenite treatment. Therefore, IBV infection does not result in the degradation of G3BP1.

### 3.2. IBV Replication Inhibits Chemical Induction of Stress Granules

**Figure 2:**
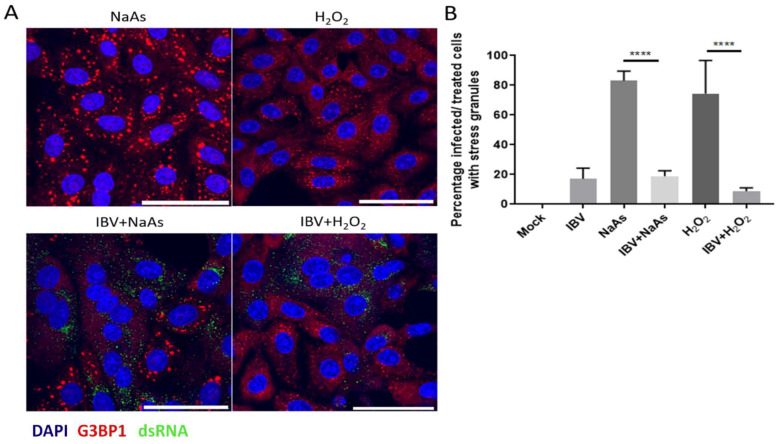
IBV inhibits eIF2α-dependent and independent stress granule induction. (**A**) Vero cells were mock infected or infected with IBV for 24 hours. Prior to fixation, cells were treated for 1 hour with 500 μM sodium arsenite (NaAs) to activate the eIF2α-dependent pathway or for 2 h with 2 µM hydrogen peroxide (H_2_O_2_) to activate the eIF2α-independent pathway. At 24 hpi, cells were fixed and stress granules (SG) were labelled with an anti-G3BP1 antibody (red). IBV infection was detected with an anti-dsRNA antibody (green). Nuclei were stained with DAPI (blue) and scale bar indicates 50 μm. (**B**) Images from (**A**) were quantified by manual counting of SG positive cells, identified by counting infected or treated cells with G3BP1 foci. A minimum of 50 cells were counted. Data from three independent replicates. Asterisks indicate statistical significance as measured by one-way ANOVA, **** *p* < 0.0001.

As IBV replication did not induce SG in every infected cell, it was hypothesised that IBV may be able to inhibit the formation of canonical SG. To test this, cells were infected with IBV for 24 h and prior to fixation, cells were treated with sodium arsenite for 1 hour or hydrogen peroxide for 2 h to induce SG formation. Sodium arsenite induces eIF2α-dependent SG by activating the eIF2α kinase HRI. Hydrogen peroxide induces SG via HRI and GCN2 [39], but also in an eIF2α independent process, by disrupting the eIF4F complex. Following fixation, cells were labelled with anti-dsRNA to detect virus infected cells and anti-G3BP1 to visualize SG. In uninfected cells, treatment with either sodium arsenite or hydrogen peroxide resulted in the formation of SG (Figure 2A). However, in IBV infected cells, both sodium arsenite and H_2_O_2_ induction of SG were blocked with G3BP1 in infected cells, remaining largely diffuse (Figure 2A). The percentage of cells containing G3BP1 foci was then determined (Figure 2B). In the absence of chemical treatment, 17% of IBV infected cells contained SG. When mock infected cells were treated with sodium arsenite or hydrogen peroxide, 83% and 74% of cells were positive for SG, respectively. However, when IBV infected cells were sodium arsenite or hydrogen peroxide treated, only 18% and 9% infected cells contained SG, respectively. Therefore, IBV infection inhibits both eIF2α-dependent and independent SG induction.

### 3.3. Stress Granules in IBV Infected Cells Are Canonical

**Figure 3:**
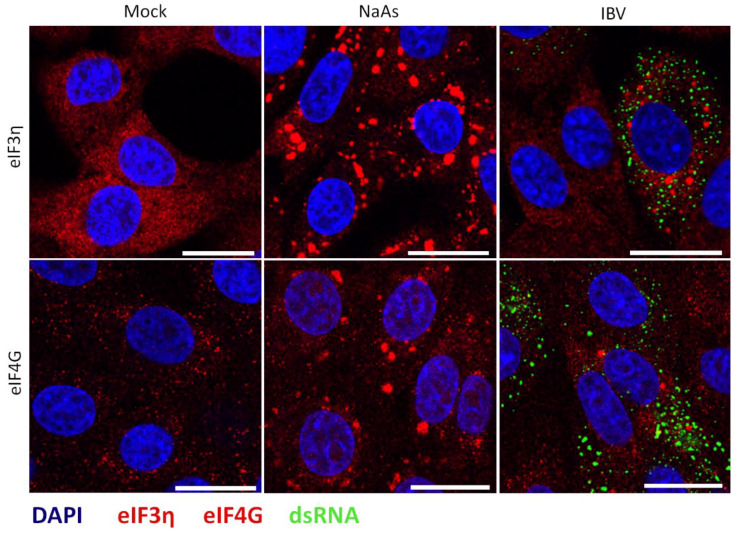
IBV induced stress granules contain multiple stress granule markers. Vero cells were mock infected or infected with IBV. One hour prior to fixation, where indicated cells were treated with sodium arsenite (NaAs). At 24 hpi, cells were fixed and labelled with dsRNA (green) and anti-eIF3η (red) or eIF4G (red), Nuclei were stained with DAPI (blue). Scale bars indicates 20 µm. Images are representative of three independent repeats.

Several viruses have been shown to promote the formation of specific virus-induced cytoplasmic foci, by recruitment and relocalisation of many SG components, including G3BP1 and G3BP2 [22,24,25,40]. Therefore, following the identification of G3BP1 puncta in some IBV infected cells, the nature of these puncta was investigated, to determine whether they were canonical SG or virus-specific granules. Canonical SG contain multiple SG markers such as G3BP1, translation initiation factors, ribosomal subunits and mRNA. Therefore, the presence of punctate translation initiation factors eIF3η and eIF4G in infected cells was investigated. Cells were infected with IBV and after 24 h, cells were fixed and labelled with anti-dsRNA and either anti-eIF3η or anti-eIF4G. As expected, eIF3η and eIF4G were diffuse within the cytoplasm in mock infected cells (Figure 3). Similar to previous observations using G3BP1, in a proportion of virus infected cells, both eIF3η and eIF4G were found in cytoplasmic puncta, with the remaining infected cells containing diffuse eIF3η or eIF4G (Figure 3). Therefore, IBV infection induces the formation of SG that contains multiple SG marker proteins. 

**Figure 4:**
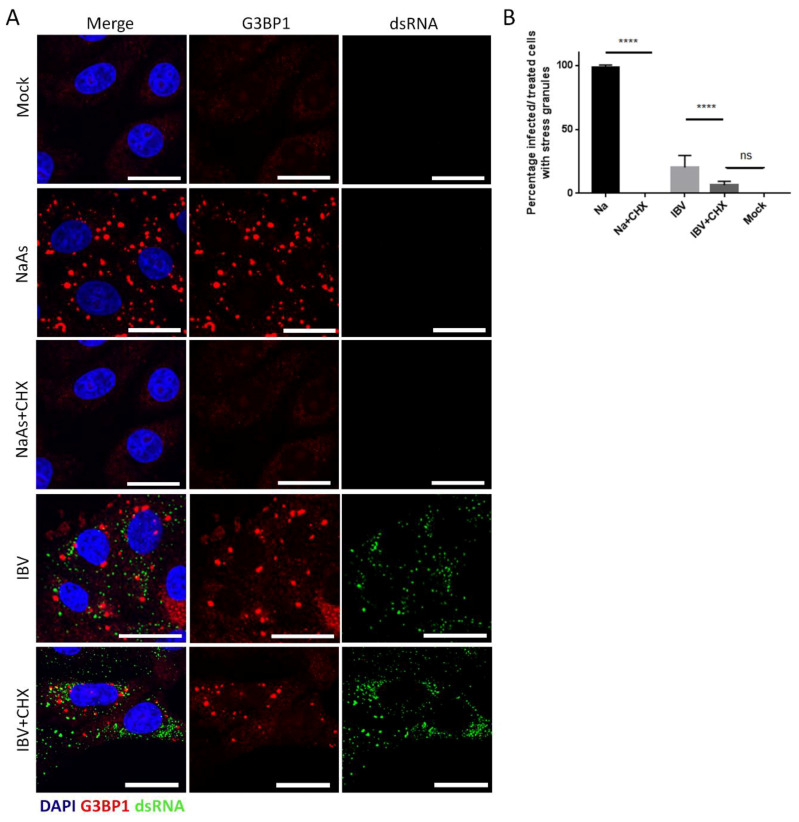
IBV induced stress granules are dissolved by cycloheximide treatment. (**A**) Vero cells were mock infected or infected with IBV. Where indicated cells were treated with sodium arsenite (NaAs). Cells were then mock treated or treated with cycloheximide (CHX) to dissolve SG. At 24 hpi, cells were fixed and labelled with anti-G3BP1 (red) to detect stress granules (SG) and IBV infected cells were detected with an anti-dsRNA antibody (green). Nuclei were stained with DAPI (blue). Scale bar indicates 20 µm. (**B**) Images from (**A**) were quantified to determine the percentage of cells containing SG. A minimum of 50 cells were counted. Mean and standard deviation of three independent replicates are shown. Asterisks indicate statistical significance, as measured by one-way ANOVA, **** *p* < 0.0001; ns, not significant.

In addition to containing multiple SG marker proteins, canonical SG are dissolved in the presence of cycloheximide. As mRNAs are constantly shuttled between SG and ribosomes, cycloheximide binding to the ribosome, preventing release of mRNA, inhibits recycling to SG. As a result, SG are dissolved. To further understand the nature of IBV induced SG, their susceptibility to cycloheximide treatment was determined. Cells were infected with IBV for 24 h, and one hour prior to fixation, cells were treated with cycloheximide. Cells were then labelled with anti-dsRNA and anti-G3BP1. Firstly, it was confirmed that SG induced with sodium arsenite in uninfected cells were dissolved by treatment with cycloheximide (Figure 4A). When cycloheximide treatment was applied to IBV infected cells, a significant decrease in the number of cells containing SG was observed. The percentage of infected cells containing SG was quantified (Figure 4B) and, interestingly, the number of IBV infected cycloheximide treated cells containing SG was reduced to a value not significantly different from mock cells. Together, this shows that IBV infection induces SG that contain multiple SG markers and are susceptible to cycloheximide, indicating that they are likely to be canonical SG.

### 3.4. IBV Genomic RNA Is not Diverted to Stress Granules during Infection

**Figure 5:**
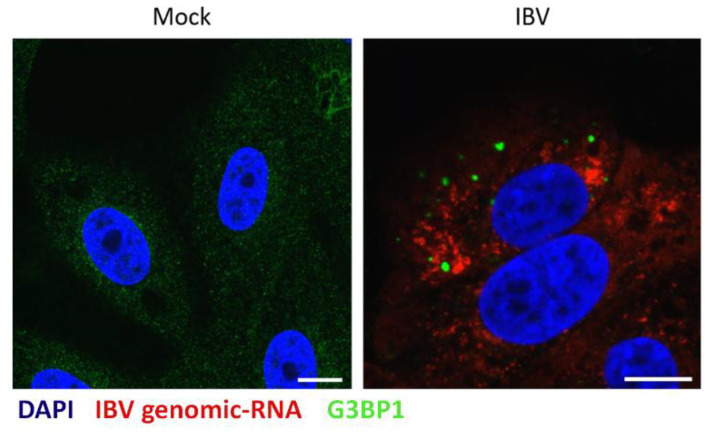
IBV genomic RNA is not diverted to stress granules during infection. Vero cells were mock infected or infected with IBV for 24 hours. IBV genomic RNA (red) was detected using Fluorescent in situ Hybridization (FISH) probes and an anti-G3BP1 antibody was used to detect SG (green). Nuclei were stained using DAPI (blue). Scale bars indicate 10 µm. Images are representative of three independent replicates.

It was previously found that during the replication of *Alphacoronavirus* TGEV, viral RNA was targeted to virus-induced SG, and this was thought to be important for their anti-viral function [26]. Therefore, to further understand IBV induced SG and to determine whether IBV RNA is also targeted to SG, viral genomic RNA was visualized using FISH. Cells were infected with IBV or mock infected. After 24 hours, cells were fixed and labelled with FISH probes specific for IBV genomic RNA and anti-G3BP1 (Figure 5). Viral genomic RNA was found to be located in foci within the cytoplasm. In addition, G3BP1 puncta were detected in a percentage of infected cells, as seen before. However, no co-localization was observed between viral genomic RNA and G3BP1 containing SG. Therefore, IBV genomic RNA is not targeted to SG.

### 3.5. Stress Granules Are not Diverted to Sites of Virus Replication

**Figure 6:**
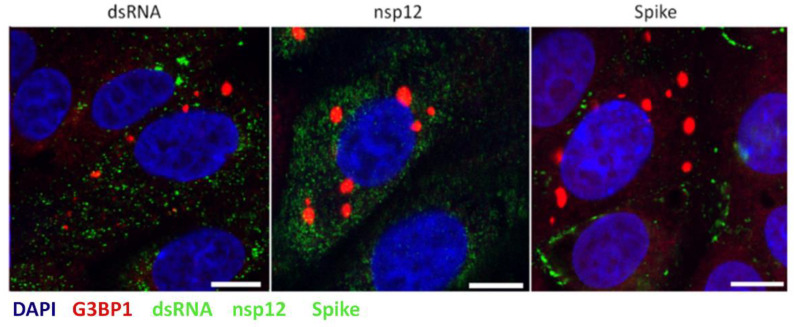
Stress granule markers are not diverted to sites of IBV replication. Vero cells were infected with IBV. At 24 hpi, cells were labelled to detect G3BP1 (red) and either dsRNA, nsp12 or spike (green). Nuclei were stained with DAPI (blue). Scale bar indicates 10 µm. Images are representative of three independent replicates.

During the replication of several other viruses including Ebola virus, West Nile virus, dengue virus and tick-borne encephalitis virus, SG markers are redirected to sites of virus replication [41,42,43]. To investigate whether IBV induced SG co-localize with sites of viral RNA synthesis or virion assembly, cells were infected with IBV and after 24 h, fixed and labelled with anti-G3BP1, as well as antibodies specific for dsRNA, thought to be an intermediate in viral RNA synthesis, nsp12, the viral RNA-dependent RNA polymerase or spike protein (anti-S2), to label sites of progeny virus assembly. Consistent with earlier experiments, dsRNA did not co-localize with G3BP1 foci (Figure 6). In addition, G3BP1 did not to co-localize with either nsp12 or spike. Therefore, IBV does not direct SG markers to sites of virus replication.

### 3.6. IBV Infection Induces A Translational Shut-Off that Increases with the Duration of Infection

**Figure 7:**
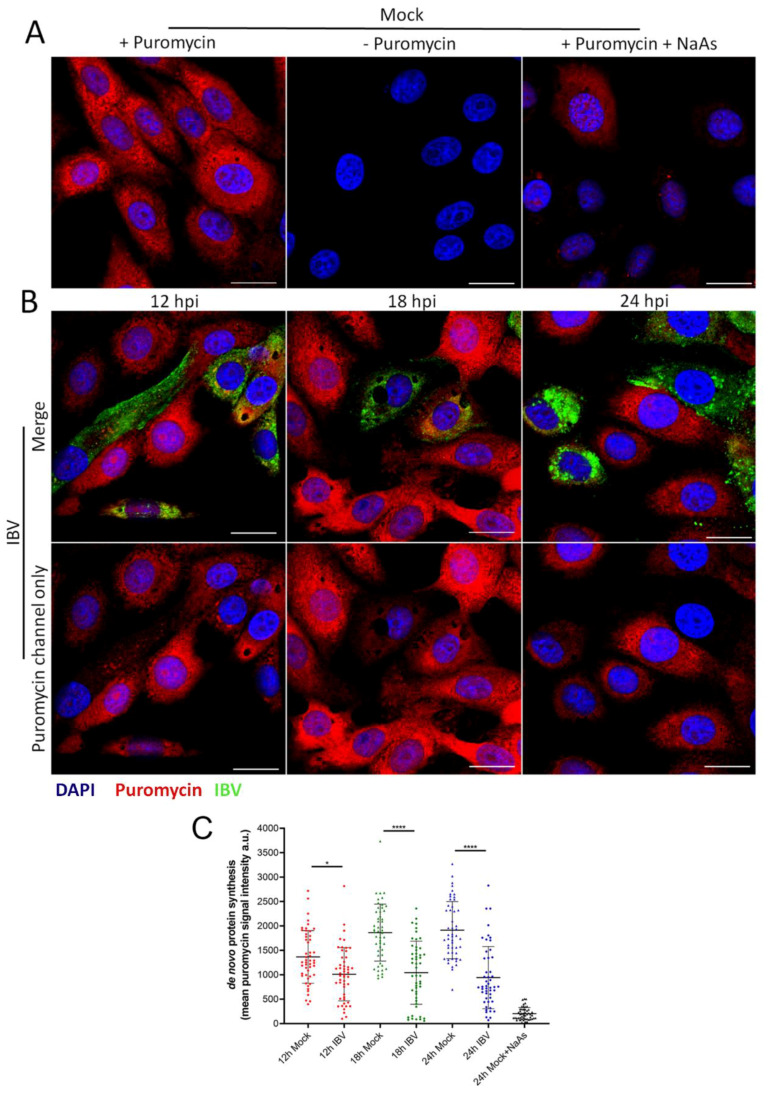
IBV infection results in translational shut-off. Vero cells were mock infected (**A**) or infected with IBV (**B**). At 23 hpi, positive control cells were treated with sodium arsenite (NaAs) to induce inhibition of translation. At 12, 18 and 24 hpi cells were treated with puromycin (or –puromycin) followed by emetine. Cells were then fixed, and nascent polypeptides were labelled with puromycin detected using an anti-puromycin antibody (red) and IBV infected cells were labelled with an anti-IBV antibody (green). Nuclei were stained with DAPI (blue). Scale bar indicates 20 µm. (**C**) Representative scatterplot of de novo protein synthesis from (**A**) and (**B**), measured by fluorescence intensity of the puromycin signal, analyzed in 50 cells. Data presented is representative of three independent replicates. Asterisks indicate statistical significance, as measured by one-way ANOVA, * *p* = 0.02; **** *p* < 0.0001.

The formation of SG in cells is closely associated with an inhibition of translation. Previous work has demonstrated that IBV replication is associated with a shut-off of host translation from around 12 hpi, with the translation of viral proteins also ceasing by 24 hpi [34]. However, translational activity on a single cell level has not been characterized. Therefore, RPM was used to visualize actively translating ribosomes over a time course of infection [37]. Cells were infected with IBV and nascent polypeptides labelled with puromycin at 12, 18 and 24 hpi, followed by the stalling of translating ribosomes with emetine. Cells were then fixed and labelled with an anti-puromycin antibody to detect nascent polypeptides and an anti-IBV antibody to detect infected cells. In mock infected cells, active translation was detected, with a diffuse puromycin signal throughout the cytoplasm. This signal was absent without puromycin treatment or upon treatment of cells with sodium arsenite to inhibit translation (Figure 7A). Following IBV infection at all three time points studied, two phenotypes were observed. Some cells contained the diffuse puromycin signal detected in mock infected cells. Alternatively, a proportion of infected cells showed reduced puromycin signal (Figure 7B). To enable the level of translational activity to be determined, puromycin signal was quantified in at least 50 infected cells and surrounding non-infected cells (Figure 7C). This indicated that there was a shut-off of translation at all time points. Furthermore, the degree of translational inhibition increased as infection progressed with a more pronounced shut-off at 18 and 24 than at 12 hpi. Therefore, consistent with previous work [34], this single cell RPM indicates that translational shut-off in infected cells is seen from 12 hpi and increases with the duration of infection.

### 3.7. Stress Granule Formation in IBV Infected Cells Is Correlated with Translational Shut-Off

**Figure 8:**
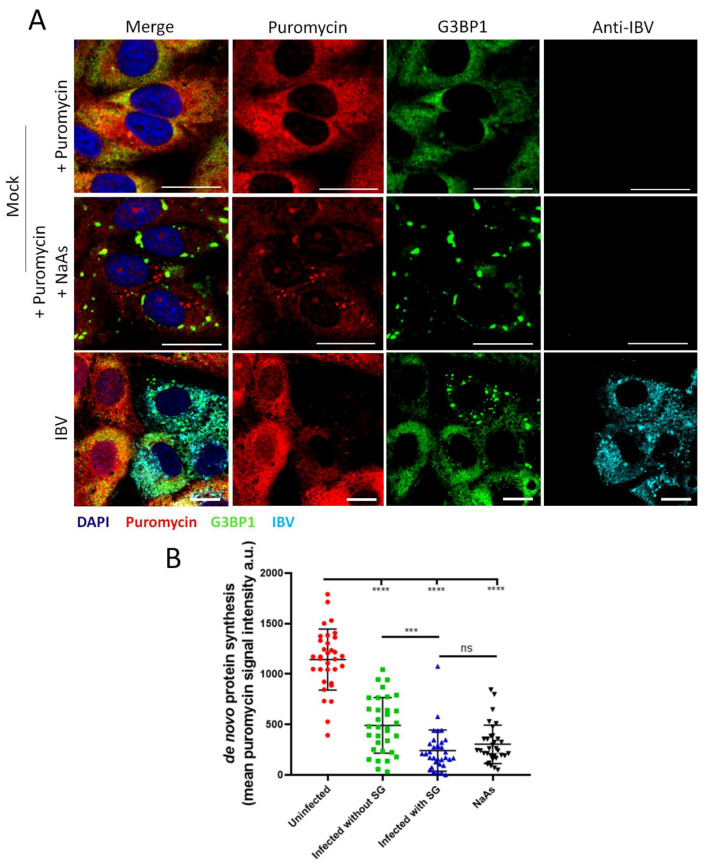
SG formation in IBV infected cells is correlated with increased translational shut-off. (**A**) Vero cells were mock infected or infected with IBV. At 23 hpi, positive control cells were treated with sodium arsenite (NaAs), to induce inhibition of translation. At 24 hpi, cells were treated with puromycin followed by emetine. Cells were then fixed, and nascent polypeptides labelled with puromycin were detected using an anti-puromycin antibody (red), stress granules labelled with anti-G3BP1 (green) and IBV infected cells labelled with an anti-IBV antibody (cyan). Nuclei were stained with DAPI (blue). Scale bar indicates 20 µm. (**B**) Representative scatterplot of de novo protein synthesis from (**A**), measured by fluorescence intensity of the puromycin signal analyzed in 80 cells. Data presented is representative of three independent replicates. Asterisks indicate statistical significance, as measured by one-way ANOVA, *** *p* = 0.0004; **** *p* < 0.0001.

As previously demonstrated, IBV induces a translational shut-off that increases over the duration of infection, with two phenotypes showing variation in the degree of translational shut-off. In addition to this, IBV induces SG in 20% of infected cells. The translational status of infected cells containing G3BP1 foci was therefore assessed to determine whether the two different translational profiles could be attributed to the presence or absence of SG. Cells were infected with IBV and then treated as before, using the RPM method. At 24 hpi cells were fixed and labelled using anti-puromycin, anti-G3BP1 and anti-IBV antibodies (Figure 8A) and the intensity of puromycin label quantified as before (Figure 8B). Active translation was detected in mock infected cells, with a diffuse puromycin signal throughout the cytoplasm. Upon stimulation with sodium arsenite, the accumulation of G3BP1 into SG correlated with a strong reduction in puromycin signal, confirming that SG formation is coupled to translational shut-off. The level of translational activity was then determined in infected cells, with and without SG. While infected cells containing SG exhibited a potent translational impairment with puromycin labelling levels similar to those detected in cells stimulated with sodium arsenite, infected cells that did not contain SG also displayed markedly reduced puromycin signal compared to uninfected cells. This demonstrates that the translational shut-off seen in IBV-infected cells cannot be solely attributed to SG formation and that translation inhibition and SG formation are uncoupled.

### 3.8. Stress Granule Formation and Translational Shut-Off during IBV Replication Are Independent of eIF2α Phosphorylation

**Figure 9:**
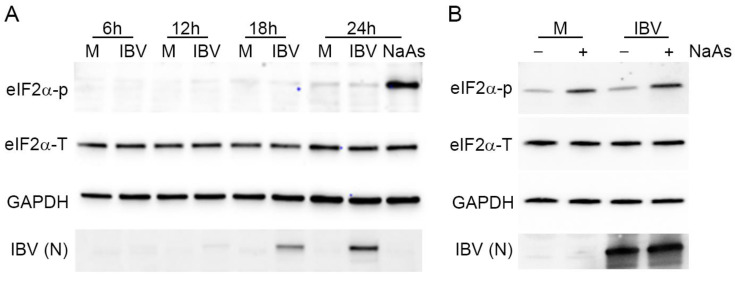
Stress granule formation and translational shut-off during IBV replication are independent of eIF2α phosphorylation. (**A**) Vero cells were mock infected or infected with IBV. At 23 hpi, cells were treated with sodium arsenite (NaAs), to induce phosphorylation of eIF2α. At 6, 12, 18 and 24 hpi, cells were washed and lysed. Samples were then separated by SDS-PAGE and transferred to nitrocellulose. Total eIF2α (eIF2α-T) was detected with anti-eIF2α, phosphorylated eIF2α (eIF2α-p) was detected with anti-eIF2α-p. IBV proteins were detected using an anti-IBV antibody, with a band corresponding to the IBV nucleocapsid protein shown (IBV (N)) and an anti-GAPDH antibody was used as a loading control. (**B**) Vero cells were mock infected or infected with IBV. At 23 hpi, where indicated, cells were treated with NaAs. Cells were lysed at 24 hpi and processed and labelled as in (**A**). Blots are representative of three independent replicates.

Both SG formation and translational shut-off are usually associated with phosphorylation of eIF2α. Previous work by others has demonstrated that IBV infection results in eIF2α phosphorylation at early time points, but that the virus inhibits this as infection progresses [32]. Therefore, the phosphorylation status of eIF2α was investigated. Vero cells were infected with IBV and lysed at 6, 12, 18 and 24 hpi. Proteins were separated by SDS-PAGE and transferred to nitrocellulose. Blots were labelled using anti-eIF2α to detect total eIF2α, anti-eIF2α-p to detect the phosphorylated eIF2α, anti-IBV to detect virus and anti-GAPDH as a loading control (Figure 9A). Total levels of eIF2α remained unchanged throughout infection. Although eIF2α phosphorylation was achieved using sodium arsenite treatment, no eIF2α phosphorylation was detected at any time point during IBV infection, with levels remaining comparable to that of mock infected cells. Subsequently, to determine whether IBV infection actively inhibits phosphorylation of eIF2α, Vero cells were infected with IBV and then treated with sodium arsenite prior to cell lysis (Figure 9B). As before, the sodium arsenite treatment of mock infected cells resulted in a significant increase in the level of phosphorylated eIF2α. When IBV infected cells were treated with sodium arsenite, there was also a significant increase in the level of phosphorylated eIF2α when compared to IBV infected untreated cells. Significantly, the level of phosphorylated eIF2α in these cells appeared comparable to that in mock infected sodium arsenite treated cells (Figure 9B). Together, this demonstrates that SG formation and translational shut-off observed during IBV replication both occur in the absence of detectable levels of eIF2α phosphorylation, but that IBV infection does not actively inhibit eIF2α phosphorylation. 

## 4. Discussion

Here, we present a study that furthers our understanding of how IBV regulates the important cellular pathways of the ISR and translation. Firstly, we have demonstrated that IBV infection inhibits both eIF2α-dependent and independent SG formation. Several other viruses, including Kaposi’s sarcoma-associated herpesvirus, Zika virus, West Nile virus and Junin virus have been shown to inhibit SG signaling via regulation of the eIF2α-dependent pathway [44,45,46,47]. These viruses achieve this by inhibiting activation of PKR [45], thereby preventing eIF2α phosphorylation [47] or by dephosphorylating eIF2α [44]. Zika virus was found to upregulate growth arrest, and DNA-damage-inducible 34 (GADD34), a component of the protein phosphatase 1 (PP1) complex, and subsequent dephosphorylation of eIF2α [44]. Interestingly, in the present study, IBV was also found to inhibit eIF2α-independent SG signaling. Flaviviruses and Ebola virus also inhibit both eIF2α-dependent and independent signaling. Ebola virus achieves this via an interaction between VP35 and several SG components including; G3BP1, eIF3 and eEF2 [41,48,49]. Whether any IBV proteins interact directly with SG components to inhibit SG formation remains to be determined. Interestingly, hydrogen peroxide has also been found to inhibit SGs via the oxidation of TIA-1. Arimoto-Matsuzaki et al. (2016) also concluded that when cells are exposed to both oxidative stress and ER stress via protein misfolding, these cells cannot form SG [50]. It is therefore possible that the inhibition of hydrogen peroxide induced SGs presented here is achieved via ER stress caused during IBV infection prior to hydrogen peroxide treatment [51].

Despite IBV regulation of multiple SG signaling pathways, infection results in the formation of SG in approximately 20% of infected cells. Numerous viruses such as poxviruses and reoviruses are known to divert SG components to sites of virus replication to benefit the virus [52,53]. However, although not exhaustive, our analysis suggests that the SG formed in 20% of infected cells are canonical SG produced in response to virus replication. IBV induced SG were found to contain multiple SG marker proteins and were susceptible to cycloheximide treatment, hallmarks of canonical SG. In addition, the SG markers did not co-localize with markers for viral RNA synthesis or particle assembly. Therefore, they do not appear to resemble the virus-specific granules produced during the replication of other viruses. This then raises the interesting question of how SG form in this subset of cells. It is possible that the viral control of SG signaling may alter over the course of infection. However, eIF2α was not phosphorylated, even at early time points post infection, and the SG positive subpopulation of cells remained constant at all time points tested. Therefore, this would not appear to be the case. Other viruses have also been shown previously to induce SG in only a proportion of infected cells. For example, Semliki Forest virus infection resulted in SG in 63% of infected cells at 4 hpi, with a further decrease after this point [54]. In chronic Hepatitis C virus (HCV) infection, an oscillation of the stress response is seen, in which 40% of HCV infected cells treated with interferon-α had SG, but in a live time course, this was shown to oscillate in a cell-specific rhythm, with 97% of cells displaying SG at some point during infection. This appears to be a strategy by HCV to modulate the cellular stress response by PKR activated eIF2α phosphorylation or conversely, dephosphorylation of eIF2α by the upregulation of GADD34 in a balancing act to prolong cell survival with oscillating stalls in translation and cell division [55]. Therefore, it is possible that in the IBV infected cells containing SG, viral regulation of eIF2α phosphorylation or other SG signaling pathways is less efficient, or these cells perhaps represent a more complex balancing act. Another possible explanation for this subset of SG displaying cells is a pre-priming of the cellular innate immunity via a paracrine signaling effect, as seen with interferon (IFN) signaling, in which paracrine IFN activates the JAK/STAT pathway and upregulates interferon stimulated genes (ISGs). Vero cells do not secrete IFN, and so are unlikely to use this specific paracrine signaling pathway, however it cannot be ruled out for other signaling molecules. Analysis at the single cell level will likely be required to tease apart the mechanism of SG formation in the subset of IBV infected cells that contain them.

Other members of the CoV family have been found to display markedly different relationships with SG and their regulation. Transmissible gastroenteritis virus (TGEV) infection was shown to induce specific antiviral SG. In contrast to observations seen here where IBV genomic RNA did not co-localize with G3BP1, these TGEV specific granules feature an interaction between polypyrimidine tract binding protein (PTB) and viral genomic and sub-genomic RNA [26]. During mouse hepatitis virus (MHV) infection, translational shut-off and SG formation was also observed and MHV replication was enhanced upon infection of eIF2αS51A^−/−^ cells or TIA^−/−^ cells in which translational inhibition and SG formation are impaired, indicating an inhibitory role for SG during MHV replication [27]. Similar to our observations here, MERS-CoV inhibits SG formation. This is achieved via an interaction between MERS-CoV accessory protein 4a and dsRNA, preventing PKR activation [31]. Therefore, taken together, and in agreement with our findings here that IBV inhibits multiple SG induction pathways, this suggests an antiviral function for SG during CoV replication.

SG usually form following translation inhibition, as a result of the aggregation of stalled mRNPs, translation initiation factors and RNA binding proteins. Therefore, the translational activity of IBV infected cells was investigated. In agreement with previous work [34], translation inhibition occurred during IBV replication from 12 hpi and the degree of inhibition increased as infection progressed. However, only 20% of infected cells contain SG and this remained constant across all time points studied. It was considered possible that cells with reduced translational activity are the 20% observed to contain SG, even though this would not account for all the cells found to have reduced levels of translation, particularly at later time points. Therefore, the relationship between SG and translational inhibition in IBV infected cells was assessed using RPM and SG staining simultaneously. This confirmed that de novo protein synthesis in IBV infected cells without SG is significantly reduced, although it is reduced to a greater extent when SG are present. Therefore, the translational inhibition seen during IBV infection cannot be solely attributed to SG formation and, interestingly, there is an uncoupling of translational arrest and SG formation. Indeed, we observe both SG formation and translational inhibition in the absence of eIF2α phosphorylation, indicating altered signaling for both pathways. This situation has also been observed during murine norovirus (MNV) replication, where cellular translation is inhibited and canonical SG assembly is blocked through the repurposing of G3BP1 independently from eIF2α phosphorylation [56,57]. MNV translational control is achieved via the phosphorylation of eIF4E by Mnk1, which in turn, is activated by the p38 kinase [58,59]. In addition, several CoV have been shown to activate the interferon-induced endoribonuclease RNase L [60,61]. RNase L cleaves cellular and viral RNAs producing ligands of pattern recognition receptors to amplify IFN production and the antiviral response [62]. Interestingly, RNase L has recently been shown to reduce translation independently of PKR, phospho-eIF2-alpha, and SGs [63]. Moreover, RNase L activity has been linked with the assembly of specific membrane-less organelles [63,64]. In cells treated with the PKR ligand poly(I:C), RNase L activation also resulted in the inhibition of chemically-induced SGs, similar to our observations with IBV, and the assembly RNase L-bodies (RLB) that are small SG-like punctate, that do not require G3BP1 for assembly and are uncoupled from translation inhibition via PKR [63]. Interestingly, another study using the specific RNase L ligand 2-5A showed that RNase L stimulation resulted in the assembly of antiviral stress granules (avSGs) [64]. These avSGs are different from the RLBs in that they require G3BP1 for assembly, which interacts with PKR and RIG-I in these granules, but also OAS and RNase L. The assembly of avSGs further stimulates IRF3-mediated IFN production, rather than IFN signaling, and may provide a platform for the interaction of RNA ligands, with pattern recognition receptors to amplify the IFN production. Despite these discrepancies, further investigation into the role of RNase L in IBV-mediated regulation of translation and SGs is warranted. The mechanism of IBV translational control is currently unknown. Other CoVs have been shown to inhibit translation through the action of a viral non-structural protein, nsp1, which binds the 40S ribosomal subunit and cleaves host mRNA [65]. However, IBV does not express nsp1. Instead, IBV accessory protein 5b was found to be responsible for translational shut-off and the stability of some mRNAs tested was actually increased upon IBV infection, suggesting a completely different mechanism for the control of cellular translation [34].

During this work, IBV replication did not result in phosphorylation of eIF2α, at any of the time points tested. Furthermore, infection was not able to limit sodium arsenite induction of eIF2α phosphorylation, showing that IBV cannot actively inhibit eIF2α phosphorylation. This is in contrast to previous findings that IBV nsp2 is a weak antagonist of PKR and that GADD34 is upregulated during IBV infection, resulting in decreased levels of phosphorylated eIF2α [32]. However, recently GADD34 has been shown to promote SG disassembly and block further stress sensing and eIF2a phosphorylation, but also to contribute to impairing SG assembly independently from the eIF2a pathway [63,66]. Therefore, we do not exclude that GADD34 induction may contribute to the absence of SGs in IBV-infected cells. A subsequent study by the same laboratory found that IBV infection also induced the phosphorylation of PERK, and the subsequent activation of ATF4 and the proapoptotic GADD153, again resulting in dephosphorylation of eIF2α [33]. The reason for the inconsistency between our current findings and the previous work is not clear, although one methodological difference in the current study is the use of sodium arsenite to induce eIF2α phosphorylation, which acts via HRI, whilst the previous studies showed the activation if eIF2α phosphorylation in virus infected cells, via activation of either PKR or PERK. Notably however, in our work presented here IBV replication did not induce phosphorylation of eIF2α at any time point, and it is therefore not necessarily surprising that mechanisms to dephosphorylate eIF2α are also not activated. Indeed, the signaling molecule required for activation of PKR, dsRNA, is known to be concealed within virus induced vesicles during coronavirus replication [67]. Furthermore, interferon signaling, which also relies upon the sensing of dsRNA is not activated in IBV infected cells until very late time points, consistent with the shielding of dsRNA from cellular detection [68]. Therefore, the activation of these various cellular signaling pathways in response to IBV infection is likely to be prevented, consistent with our findings.

## 5. Conclusions

In the present study, we have demonstrated that IBV replication effectively blocks both eIF2α-dependent and eIF2α-independent SG signaling pathways. In addition, IBV replication results in a shut-off of translation. However, interestingly, in a proportion of infected cells, canonical SG are formed that do not localize with sites of viral replication and do not contain viral RNA. This raises the interesting future possibility of being able to study the composition and function of canonical cellular SG in virus infected cells. In addition to these findings, in IBV infected cells, both translational repression and SG formation were found to occur in the absence of eIF2α phosphorylation, although IBV replication was not able to actively inhibit eIF2α phosphorylation. Therefore, the IBV infection of cells results in a dysregulation and uncoupling of several important cellular signaling pathways. The mechanism behind this dysregulation remains to be determined, but we have furthered our understanding of how IBV changes the cellular environment to make it favorable for virus replication. 

## Supplementary Materials

The following are available online at https://www.mdpi.com/1999-4915/12/5/536/s1, Figure S1. Active replication is required for IBV induced stress granules.
