# Supplementary material for: Infectious Bronchitis Virus Regulates Cellular Stress Granule Signaling
Source: Viruses. 2020 May 14;12(5):536. doi: 10.3390/v12050536 (PMC7291021; doi:10.3390/v12050536)
Supplement: Supplementary file 1 [file viruses-12-00536-s001.pdf]

Supplementary figures

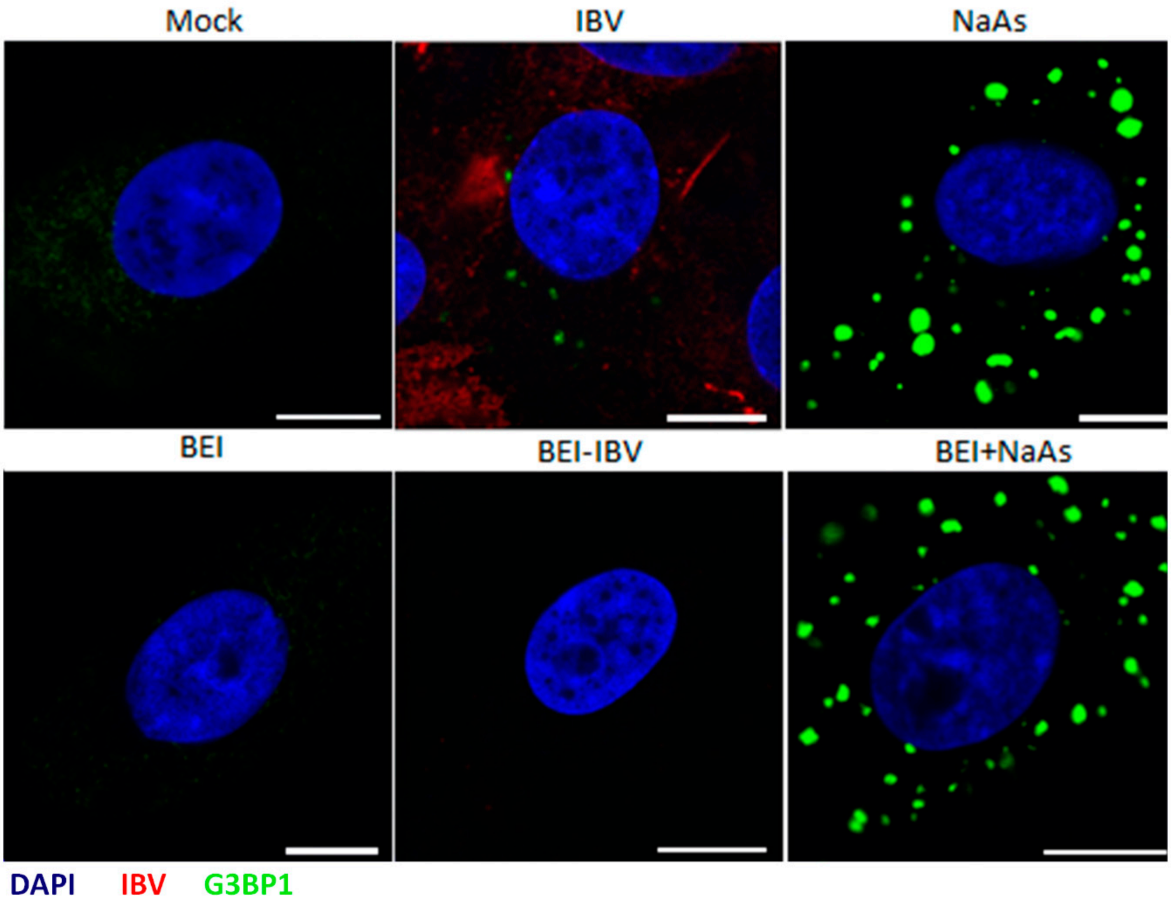

Figure S1. Active replication is required for IBV induced stress granules. Vero cells were mock infected or infected with IBV or BEI-inactivated IBV. Mock conditions using BEI-inactivation reagents with and without sodium arsenite were used in isolation from virus to ensure these did not alter SG formation. Cells were labeled with anti-G3BP1 (green) and anti-IBV (red). Nuclei were stained with DAPI (blue). Scale bar indicates 10 μm.

© 2020 by the authors. Licensee MDPI, Basel, Switzerland. This article is an open access article distributed under the terms and conditions of the Creative Commons Attribution (CC BY) license (http://creativecommons.org/licenses/by/4.0/).
